# A Pseudovirus-Based Entry Assay to Evaluate Neutralizing Activity against Respiratory Syncytial Virus

**DOI:** 10.3390/v15071548

**Published:** 2023-07-14

**Authors:** Longbo Hu, Jiajing Jiang, Yongjie Tang, Lingling Mei, Liping Wu, Leyi Li, Hongzhou Chen, Fei Long, Jing Xiao, Tao Peng

**Affiliations:** 1 State Key Laboratory of Respiratory Disease, Sino-French Hoffmann Institute, School of Basic Medical Science, Guangzhou Medical University, Guangzhou 511436, China; longbo_hu@aliyun.com (L.H.); jiajingj825@163.com (J.J.); ling_ling_mei@163.com (L.M.); gdpu_wlp@163.com (L.W.); llyclubs@163.com (L.L.); visunzchen@hotmail.com (H.C.); long_fei78@hotmail.com (F.L.); 18520126506@163.com (J.X.); 2 Guangdong South China Vaccine Co., Ltd., Guangzhou 510663, China; 3 Greater Bay Area Innovative Vaccine Technology Development Center, Guangzhou International Bio Island Laboratory, Guangzhou 510005, China

**Keywords:** respiratory syncytial virus (RSV), pseudovirus, neutralization antibody, entry assay, vaccine

## Abstract

Respiratory syncytial virus (RSV) infection can cause life-threatening pneumonia and bronchiolitis, posing a significant threat to human health worldwide, especially to children and the elderly. Currently, there is no specific treatment for RSV infection. The most effective measures for preventing RSV infection are vaccines and prophylactic medications. However, not all population groups are eligible for the approved vaccines or antibody-based preventive medications. Therefore, there is an urgent need to develop novel vaccines and prophylactic drugs available for people of all ages. High-throughput assays that evaluate the efficacy of viral entry inhibitors or vaccine-induced neutralizing antibodies in blocking RSV entry are crucial for evaluating vaccine and prophylactic drug candidates. We developed an efficient entry assay using a lentiviral pseudovirus carrying the fusion (F) protein of type A or B RSV. In addition, the essential parameters were systematically optimized, including the number of transfected plasmids, storage conditions of the pseudovirus, cell types, cell numbers, virus inoculum, and time point of detection. Furthermore, the convalescent sera exhibited comparable inhibitory activity in this assay as in the authentic RSV virus neutralization assay. We established a robust pseudovirus-based entry assay for RSV, which holds excellent promise for studying entry mechanisms, evaluating viral entry inhibitors, and assessing vaccine-elicited neutralizing antibodies against RSV.

## 1. Introduction

Human respiratory syncytial virus (RSV) is an enveloped, single-stranded, negative-sense RNA virus that belongs to the genus *Orthopneumovirus* and the family *Pneumoviridae* [1]. The genome of RSV is approximately 15.2 kb and encodes 11 proteins, including the non-structural proteins (NS1, NS2), nucleocapsid (N), phosphoprotein (P), matrix (M), small hydrophobic surface protein (SH), transcriptional regulators (M2–1 and M2–2), polymerase (L), attachment (G), and fusion (F) glycoproteins [2]. RSV is classified into two subtypes, A and B, which circulate alternately during different seasons [3]. Within the RSV-A and RSV-B subtypes, RSV is further classified into different genotypes based on genetic variations in the G glycoprotein [4]. RSV infection is the leading cause of acute lower respiratory tract infection (ALRI) in infants and children and the second most common infectious cause of infant mortality globally [5]. Each year, around 33 million children under 5 develop ALRI due to RSV, and 3.6 million require hospitalization [6]. Furthermore, RSV infection can lead to severe illness in older and immunosuppressed adults. In 2015, an estimated 1.5 million cases of acute respiratory illness associated with RSV occurred in older adults, with approximately 14.5% of these cases resulting in hospitalization [7,8]. In addition to causing high rates of illness and death, the RSV pandemic also carries a significant economic burden [9]. However, no specific therapeutic options are currently available for treating RSV infection. Supportive care remains the primary treatment for this condition. Considerable efforts are underway to develop effective vaccines and prophylactic medications to prevent RSV infection and reduce the disease burden [10,11,12]. Currently, two vaccines, Arexvy and Abrysvo, and two antibody drugs, palivizumab and nirsevimab, have been approved for preventing RSV infection [13,14,15]. However, both vaccines are designed for older adults (over 60 years old), and there is currently no RSV vaccine available for children [16]. Palivizumab is limited to preventing severe RSV illness in high-risk infants and children. Furthermore, the high cost of antibody-based drugs like Palivizumab restricts their widespread use [17,18]. Therefore, there is a pressing need to develop broad-spectrum RSV vaccines or antivirals that can be used across diverse populations.

The entry of RSV into host cells is the initial step of the viral life cycle that leads to productive infection. As a result, it is the primary target of vaccines and prophylactic drugs. The strategy behind vaccines and prophylactic drugs is to neutralize RSV entry by vaccine-induced antibodies or antibody-based products [19]. Thus, the ability of vaccine-induced antibodies to block RSV entry is a crucial factor in evaluating the effectiveness of anti-RSV vaccines. RSV entry consists of two main steps: the attachment of the virus to its host cell, which is mediated by the G protein, and the subsequent fusion of the viral envelope with the cell plasma membrane, which is mediated by the F protein. The G protein promotes virus attachment to cell surfaces by interacting with host cell attachment factors, such as glycosaminoglycans and the fractalkine receptor CX3C-chemokine receptor 1 [20]. After attachment, the F protein interacts with cell surface receptors, including insulin-like growth factor receptor 1 (IGF1R), intercellular adhesion molecule-1 (ICAM-1), epidermal growth factor receptor (EGFR), and nucleolin (NCL). It facilitates membrane fusion by undergoing a transition from a metastable prefusion (pre-F) conformation to a stable postfusion (post-F) conformation [19,21]. Studies have shown that prefusion F induces higher levels of neutralizing antibodies than postfusion F [22,23]. Since the F and G proteins play a crucial role in RSV entry, they are the primary targets for neutralization through antibodies [24,25]. The G protein displays moderate sequence diversity, which serves as the foundation for RSV genotype classification.

In contrast, the F protein exhibits high conservation among strains and elicits broad-spectrum cross-protective immunity, rendering it a promising target for developing vaccines or antivirals [26,27,28,29]. It is worth noting that high levels of neutralizing antibodies are associated with protection against RSV infection and less severe clinical outcomes [24,30]. Therefore, measuring neutralizing antibodies is essential for evaluating an individual’s immune status and predicting clinical outcomes.

The plaque reduction neutralization test (PRNT) is widely regarded as the “gold standard” for detecting and quantifying neutralizing antibodies. However, it is a laborious and challenging process to conduct in large quantities. The pseudovirus, based on vesicular stomatitis virus (VSV) or HIV-1 virions pseudotyped with viral envelope proteins of interest, is a powerful alternative approach that can mimic the entry process of authentic viruses [31,32,33]. Pseudovirus-based entry assays have been widely used to elucidate viral invasion mechanisms and assess neutralizing antibodies against SARS-CoV-2, MERS-CoV, and HCV [32,34,35,36,37]. However, fewer entry assays based on pseudoviruses are available for detecting neutralizing antibodies against RSV.

In this study, we developed a successful entry assay for two subtypes of RSV using a lentiviral backbone with a luciferase reporter gene. The assay was based on pseudovirus and proved to be effective. Furthermore, this assay examined the efficacy of convalescent sera and fusion inhibitors in blocking RSV entry. The results obtained from the pseudovirus-based assays showed a strong correlation with those obtained from the authentic virus, indicating that the assay has great potential for evaluating vaccines and antivirals against RSV.

## 2. Materials and Methods

### 2.1. Cells and Reagents

HEK293T, Huh7.5.1, Vero, and HeLa cells were maintained in Dulbecco’s Modified Eagle Medium Hight Glucose (Gibco, Waltham, MA, USA) supplemented with 100 μg/mL streptomycin, 100 units/mL penicillin (Gibco, Waltham, MA, USA), and 10% fetal bovine serum (FBS) (ExCell Bio, Shanghai, China). The 293T-hACE2 cell line stably expressing human angiotensin-converting enzyme 2 (ACE2) was generated by lentiviral transduction of HEK293T cells with human ACE2, as previously described [38,39]. HEp-2 cells were maintained in RPMI 1640 (Gibco, Waltham, MA, USA) supplemented with 100 μg/mL streptomycin, 100 units/mL penicillin, and 10% FBS. RSV entry inhibitors, TM353121, AK 0529, and RV521, were purchased from MedChemExpress (Monmouth Junction, NJ, USA). Anti-VSV-G antibody [8G5F11] was purchased from Kerafast (Boston, MA, USA). The mouse anti-RSV-F serum and mouse anti-SARS-CoV-2 spike serum were prepared in our laboratory [39].

### 2.2. Plasmid Construction

Codon-optimized full-length F, G, and SH genes from the Long strain (GenBank: AY911262.1) [40], A2 strain (GenBank: KT992094.1), and B1 strain (GenBank: AF013254.1) were synthesized and cloned into the pCAGGS vector. All constructed plasmids were verified by sequencing.

### 2.3. Production of Pseudoviruses

Lentivirus-based pseudoviruses were prepared as previously described [39]. In brief, HEK293T cells were co-transfected with a construct encoding the viral envelope protein (F protein of RSV, G protein of VSV, or spike protein of SARS-CoV-2), a lentiviral transfer plasmid (pWPXL) expressing firefly luciferase reporter protein, and a lentiviral packaging plasmid (pSPAX2) using polyethyleneimine (PEI MAX 40k, Polysciences, Inc., Warrington, PA, USA). The supernatants were refreshed with prewarmed DMEM at 8 h post-transfection and harvested at 48 h post-transfection, followed by passage through a 0.45 μm filter and stored at −80 °C. VSV and wild-type (WT) SARS-CoV-2 pseudoviruses were generated as previously described [39].

### 2.4. Titration of RSV Pseudovirus

Huh7.5.1 cells were seeded in 96-well plates one day before titration and then infected with a series of 5-fold dilutions of RSV pseudovirus (8 duplicate wells per dilution). After 48 h of incubation, cell luminescence was measured using the Bright-Glo Luciferase Assay with a GloMax Discover luminometer (Promega, Madison, WI, USA). Non-infected cells were used as controls. Positive wells were defined as those with relative luminescence units (RLU) tenfold higher than the control. The 50% tissue culture infectious dose (TCID_50_) was calculated using the Reed-Muench method.

### 2.5. Pseudovirus-Based Neutralization Assay

Rabbit anti-RSV F serum, mouse anti-SARS-CoV-2 spike serum, and anti-VSV-G antibody were initially diluted at 1:200, followed by a 10-fold serial dilution for anti-RSV F serum and anti-VSV-G antibody, or a 2-fold serial dilution for anti-SARS-CoV-2 spike serum, and then mixed with 250 TCID_50_ pseudovirus. After incubation for 30 min at 37 °C, the mixture was added to Huh7.5.1 cells (for VSV and RSV pseudoviruses) or 293T-hACE2 cells (for SARS-CoV-2 pseudovirus) and incubated for an additional 36 h. Luminescence was measured using the Bright-Glo Luciferase Assay (Promega, Madison, WI, USA) according to the manufacturer’s instructions. The percentage of inhibition was calculated by determining the relative reduction in RLU compared to the control group (pseudovirus-infected cells without sera or antibodies). Dose-response curves were generated using a three-parameter nonlinear regression in GraphPad Prism 8.

### 2.6. Statistical Analysis

All experiments were performed at least three times. The data were presented as mean ± SD. Statistical significance was analyzed by Student’s *t* test or one-way ANOVA statistical tests where appropriate using GraphPad Prism 8. *p* < 0.05 was considered statistically significant. (* *p* ≤ 0.05, ** *p* ≤ 0.005, *** *p* ≤ 0.0005, **** *p* ≤ 0.0001).

## 3. Results

### 3.1. Optimization of the RSV Pseudovirus Packaging System

**Figure 1:**
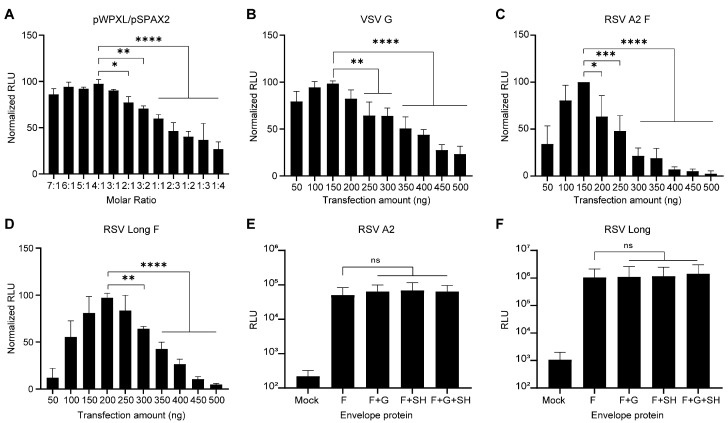
Optimization of RSV pseudovirus packaging system. (**A**) HEK293T cells were transfected with the plasmid expressing VSV-G (pMD2.G) and different molar ratios of transfer plasmid (pWPXL) and packaging plasmid (pSPAX2) as indicated. Supernatants containing pseudovirus were collected 48 h after transfection and used to infect Huh7.5.1 cells, and luciferase activity was measured 48 h after infection. (**B**–**D**). HEK293T cells in six-well plates were transfected with packaging and shuttle plasmids at a molar ratio of 4:1, as well as different amounts of plasmids expressing VSV-G (**B**), F protein of RSV A2 (**C**), or F protein of RSV Long (**D**). Supernatants containing pseudovirus were collected 48 h after transfection and used to infect Huh7.5.1 cells, and luciferase activity was assayed 48 h after infection. Each relative light unit (RLU) value was divided by the highest RLU value to normalize the luminescence. (**E**,**F**). Huh7.5.1 cells were infected with RSV pseudoviruses generated by transfecting HEK293T cells with pWPXL, pSPAX2, and plasmids encoding F with or without G and SH proteins of RSV A2 (**E**) or RSV Long (**F**), and the luciferase activity was measured at 48 h after infection. Mock cells were co-transfected with pWPXL and pSPAX2 without the envelope expression plasmid. Data are represented as means ± SD of three independent repeated experiments. *, *p* < 0.05; **, *p* < 0.01; ***, *p* < 0.001, ****, *p* < 0.0001; ns, not significant.

Three glycoproteins, namely fusion (F), attachment (G), and small hydrophobic (SH), are expressed on the envelope of the RSV virion. The G protein mediates the attachment to the host cells, and the F protein mediates subsequent virus-cell membrane fusion. As the F protein plays a crucial role in RSV entry, we opted to package the RSV pseudovirus with the F protein. Lentiviral particles were generated through the transient cotransfection of HEK293T cells with an envelope protein-expressing plasmid, a packaging plasmid (psPAX2), and a transfer plasmid (pWPXL) encoding firefly luciferase. To attain high titers of pseudoviruses, we initially determined the ideal transfection molar ratios of transfer plasmid and packaging plasmid to be 4:1, using the VSV pseudovirus as a model (Figure 1A). Next, we determined the optimal transfection amount for plasmids encoding envelope proteins at a 4:1 molar ratio of pWPXL to psPAX2. As shown in Figure 1B–D, the most effective molar ratios for transfection to produce VSV, RSV A2, and RSV Long pseudoviruses using shuttle plasmid, packaging plasmid, and envelope-expressing plasmid are 4:1:1.7, 4:1:1.5, and 4:1:2.1, respectively. Increasing the transfection amount of the envelope plasmid didn`t necessarily result in a higher titer of the pseudovirus, suggesting that the appropriate expression level of the envelope protein is the key to successful pseudovirus packaging.

### 3.2. Optimization of Thawing Temperature of RSV Pseudovirus

**Figure 2:**
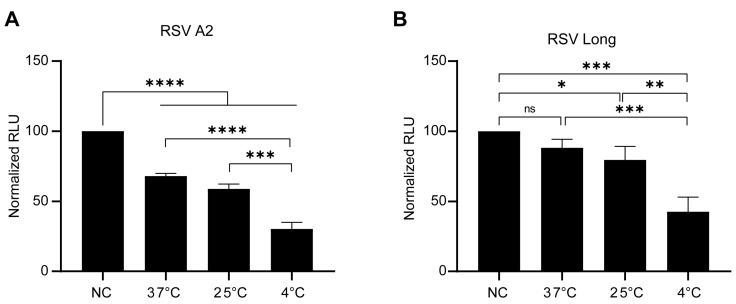
Optimization of the thawing temperature of RSV pseudovirus. RSV A2 (**A**) or RSV Long (**B**) pseudoviruses were kept on ice (no freeze-thaw control, NC) or stored at −80 °C before thawing at different temperatures and then infected Huh7.5.1 cells. Luciferase activity was measured 48 h after infection. Data are represented as means ± SD of three independent repeated experiments. *, *p* < 0.05; **, *p* < 0.01; ***, *p* < 0.001, ****, *p* < 0.0001; ns, not significant.

Previous studies have shown that elevated temperatures can induce conformational changes in the RSV F protein, thereby affecting the efficiency of RSV infection [41,42]. RSV pseudoviruses were aliquoted and stored at −80 °C before use and underwent a single freeze-thaw cycle. To maintain the highest possible pseudovirus titer, we investigated the impact of various thawing temperatures on the infectivity of RSV pseudovirus. As shown in Figure 2, the infectivity of RSV A2 and Long pseudoviruses decreased the least when the frozen pseudovirus was thawed at 37 °C, while thawing the pseudovirus at a lower temperature significantly reduced its infectivity.

### 3.3. Optimization of Key Parameters in Pseudovirus-Based Neutralization Assay against RSV

**Figure 3:**
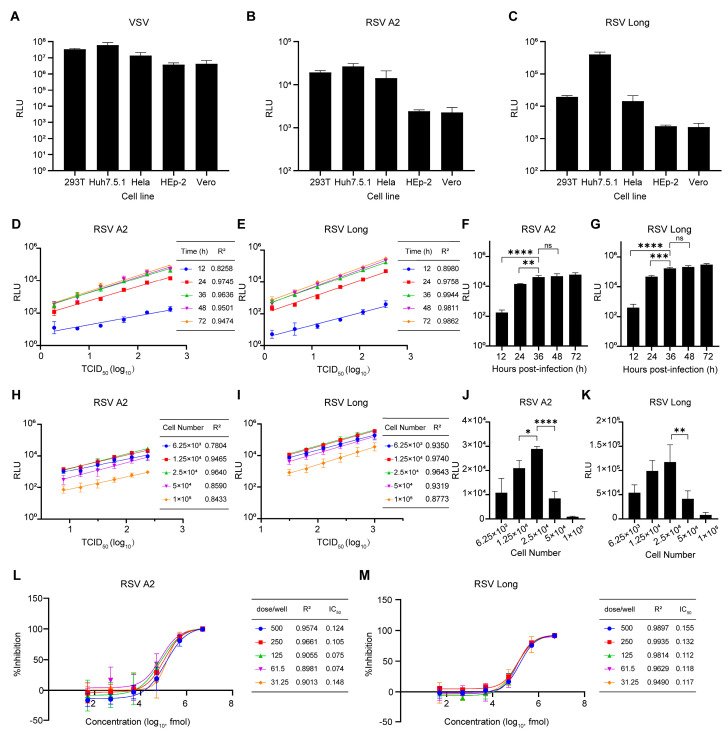
Optimization of key parameters for neutralization assay. (**A**–**C**). HEK293T, Huh7.5.1, HeLa, Hep−2, and Vero cells were infected with VSV pseudovirus (**A**), RSV A2 pseudovirus (**B**), and RSV Long pseudovirus (**C**) and luciferase activity was measured at 48 h after infection. (**D**,**E**). Huh7.5.1 cells were infected with RSV A2 pseudovirus (**D**) or RSV Long pseudovirus (**E**) at different titers, and luciferase activity was measured at different time points as indicated. Time points of detection and corresponding R^2^ values were listed in the tables. (**F**,**G**). Luciferase activity of Huh7.5.1 cells infected with RSV A2 pseudovirus (**F**) or RSV Long pseudovirus (**G**) at 400 TCID_50_/well at indicated time points. (**H**,**I**). Huh7.5.1 cells with different cell numbers were infected with RSV A2 (**H**) or RSV Long (**I**) pseudoviruses at different titers, and luciferase activity was measured 36 h after infection. Cell numbers and corresponding R^2^ values were listed in the tables. (**J**,**K**). The luciferase activity of Huh7.5.1 cells with different cell numbers infected with RSV A2 (**J**) or RSV Long (**K**) pseudoviruses at 250 TCID_50_/well. (**L**,**M**). The neutralizing activity of TMC353121 against RSV was assessed by RSV A2 (**L**) and RSV Long (**M**) pseudovirus with a dose ranging from 31.25 to 500 TCID_50_/well. Virus doses, corresponding R^2^ values, and IC_50_ were listed in the tables. Data are represented as means ± SD of three independent repeated experiments. *, *p* < 0.05; **, *p* < 0.01; ***, *p* < 0.001, ****, *p* < 0.0001; ns, not significant.

To establish an efficient and convenient pseudovirus-based neutralization assay for RSV, we optimized four critical parameters involved in this assay: cell types, detection time points, cell numbers, and virus inoculum. We initially assessed the infectivity of VSV, RSV A2, and Long pseudoviruses in various target cells, such as HEK293T (human embryonic kidney cells), Huh7.5.1 (human hepatocellular carcinoma cells), HeLa (human cervical carcinoma cells), HEp-2 (human laryngeal carcinoma cells), and Vero (African green monkey kidney cells). Susceptibility to VSV pseudovirus was similar among all cell lines, as expected (Figure 3A). However, the best susceptibility to RSV A2 and Long pseudoviruses was observed in Huh7.5.1 cells. Thus Huh7.5.1 was identified as the ideal cell line for the pseudovirus-based neutralization assay against RSV (Figure 3B,C).

To determine the optimal time point for detection, we measured luciferase activity at various time points after pseudovirus infection of Huh7.5.1 cells. We analyzed the correlation using Pearson’s correlation coefficient (R^2^) and a linear regression model. Luciferase activity strongly correlated with RSV pseudovirus titers at 36 h post-infection, with R^2^ values of 0.9636 and 0.9944 for RSV A2 and Long pseudovirus, respectively (Figure 3D,E). In addition, at 36 h post-infection compared to 24 h post-infection, longer incubation did not result in a significant increase in luciferase activity (Figure 3F,G). Therefore, we measured the luciferase activity at 36 h post-infection.

We next investigated the optimal number of Huh7.5.1 cells for RSV pseudovirus infection in the pseudovirus-based neutralization assay. Huh7.5.1 cells at different cell numbers (ranging from 6.25 × 10^3^ to 1 × 10^5^ cells/well) were infected with RSV pseudoviruses, then the luciferase activity was measured, and the linear correlation coefficient was analyzed. The highest linear correlation coefficient for RSV A2 and RSV Long pseudovirus was obtained from 2.5 × 10^4^ cells/well and 1.25 × 10^4^ cells/well, respectively (Figure 3H,I). However, the highest luminescence signals for both A2 and Long pseudovirus were obtained from 2.5 × 10^4^ cells/well, while further inoculation of more cells resulted in a significant decrease in the luminescence signal. Based on these findings, we chose 2.5 × 10^4^ cells/well for all subsequent experiments (Figure 3J,K).

The optimal viral inoculum for the pseudovirus-based neutralization assay against RSV was then tested. The neutralizing capacity of TMC353121 [43], a potent RSV fusion inhibitor, was assessed against the RSV pseudovirus using a dose range of 31.25 to 500 TCID_50_/well. Figure 3L,M shows that the highest linear correlation coefficients for RSV A2 and Long pseudoviruses were obtained when the virus was inoculated at 250 TCID_50_/well. Therefore, the 250 TCID_50_/well viral inoculation was selected as the optimal dose. A similar strategy was utilized to establish a pseudovirus-based neutralization assay for the RSV B1 strain.

### 3.4. Validation of the RSV Pseudovirus-Based Neutralization Assay

**Figure 4:**
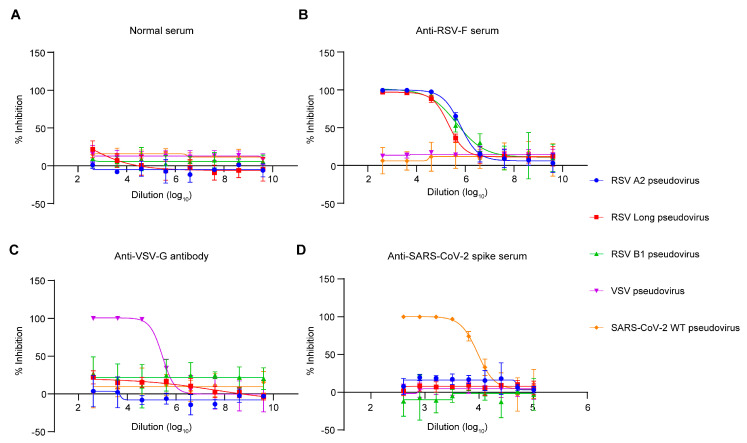
Specificity of the RSV pseudovirus−based neutralization assay. The neutralizing activity of normal mouse serum (**A**), mouse anti-RSV-F serum (**B**), mouse anti−VSV−G antibody (**C**), and mouse anti-SARS-CoV-2 spike serum (**D**) against VSV, RSV A2, RSV Long, RSV B1, and SARS-CoV-2 was evaluated using pseudovirus-based neutralization assays, respectively. Data are represented as means ± SD of three independent repeated experiments.

After optimizing the critical parameters of the pseudovirus-based neutralization assay, we next verified the specificity of this assay. We used the pseudovirus-based neutralization assay to evaluate the neutralizing ability of mouse anti-RSV-F serum, mouse anti-SARS-CoV-2 spike serum, mouse anti-VSV-G antibody, and normal mouse serum as a control against VSV, RSV, and SARS-CoV-2 pseudoviruses, respectively. As shown in Figure 4, anti-VSV-G antibody and anti-SARS-CoV-2 spike sera also exhibited activity to neutralize VSV and SARS-CoV-2 pseudoviruses specifically. In contrast, mouse anti-RSV-F serum demonstrated the ability to specifically neutralize three RSV pseudoviruses in this assay, indicating that this assay can evaluate the neutralizing capacity against RSV infection.

**Figure 5:**
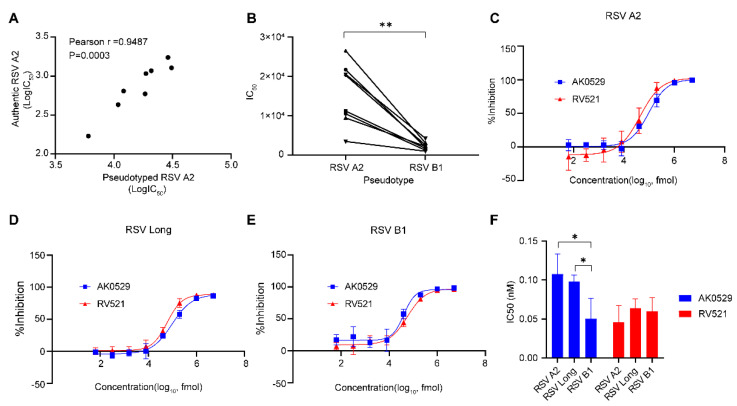
Validation of the RSV pseudovirus-based neutralization assay. (**A**). Analyses of linear correlations of IC_50_ values of eight healthy human sera between the RSV A2 pseudovirus−based neutralization assay and the authentic RSV A2 virus−based neutralization assay. These linear correlation analyses used Pearson’s correlation coefficients calculated with GraphPad Prism. (**B**). Neutralizing antibody titers (serum dilution IC_50_) of eight healthy human sera against RSV A2 and B1 were obtained from the RSV pseudovirus−based neutralization assay. (**C**–**E**). The neutralizing activity of AK0529 and RV521 against RSV A2 (**C**), RSV Long (**D**), and RSV B1 (**E**) was evaluated using pseudovirus−based neutralization assays, respectively. (**F**). Neutralizing antibody titers of AK0529 and RV521 against RSV A2, Long, and B1 were obtained from the RSV pseudovirus−based neutralization assay. Data are represented as means ± SD of three independent repeated experiments. *, *p* < 0.05, **, *p* < 0.01.

Since this assay is intended to evaluate the neutralizing capacity of RSV-related serum samples and RSV entry inhibitors, we first verified this assay with a cohort of healthy adult volunteer sera. We assessed the neutralizing ability of sera from eight healthy adults against RSV A2 using the neutralization assays based on RSV A2 pseudovirus or authentic RSV A2 virus, respectively. We compared the IC50 obtained by the two assays. We observed a strong positive correlation (Pearson r = 0.9487, *p* = 0.0003) between the pseudovirus- and live virus-based neutralization assays, suggesting the utility of this RSV pseudovirus-based neutralization assay (Figure 5A). Additionally, we compared the ability of these sera to neutralize RSV A2 and B1 using this pseudovirus-based assay. We found that these sera had a significantly higher ability to neutralize RSV A2 than RSV B1 (Figure 5B). And then, the neutralizing capacity of two RSV entry inhibitors, AK0529 and RV521 [44,45], against RSV A2, Long, and B1 were evaluated using the RSV pseudovirus-based neutralization assay. Both inhibitors showed the potent ability to neutralize RSV A2 (Figure 5C), Long (Figure 5D), and B1 (Figure 5E), suggesting that they have a broad-spectrum ability to inhibit RSV infection.

To evaluate the performance of these assays for high-throughput screening (HTS), we calculated the Z-factor using the mean and the standard deviations of the negative and positive controls [46]. A Z-factor between 0.5 and 1 represents a reliable HTS assay. The Z-factors of the RSV A2, Long, and B1 neutralization assays were 0.58, 0.7, and 0.63, respectively, when AK0529 was used as the positive control. The results indicated that the RSV pseudovirus-based neutralization assays are suitable for high-throughput screening.

Taken together, we established a robust and reliable pseudovirus-based entry assay that could be used to evaluate vaccine and therapeutic candidates targeting RSV entry.

## 4. Discussion

Serum levels of neutralizing antibodies against RSV are correlated with the prevalence and severity of RSV-related hospitalizations and protection against RSV [44,45,46]. Therefore, the level of neutralizing antibodies induced by vaccines is a crucial indicator for vaccine evaluation [47]. In addition, the neutralizing ability is a direct indicator for evaluating antivirals targeting viral entry. Therefore, the establishment of a high-throughput neutralization assay is critical for the development of vaccines and antivirals. In this study, we developed and optimized a convenient pseudovirus-based neutralization assay that enables high-throughput assessment of neutralization capacity against specific RSV-A (A2 and Long) and RSV-B (B1) subtypes.

Furthermore, the consistency of neutralization results between the live virus-based assay and the pseudovirus-based assay suggests that the latter can serve as a dependable substitute for the authentic virus-based assay in assessing neutralization efficacy. Since RSV pseudoviruses accurately mimic the entry process of authentic RSV without involving other stages of the virus life cycle. This makes them valuable tools for focusing solely on and investigating entry mechanisms, including identifying virus receptors, studying cell tropism, and revealing viral entry dynamics.

Although the conventional plaque reduction neutralization test (PRNT) is widely accepted as the gold standard for measuring neutralizing antibodies to RSV, it is a labour-intensive and time-consuming process, which makes it challenging to implement on a large scale [48]. Neutralization assays that rely on recombinant RSV viruses with reporter genes enable high-throughput detection. However, these assays necessitate advanced reverse genetics systems and specific construction strategies for different recombinant viruses [49,50,51,52]. Pseudoviruses, which present the envelope protein of the virus of interest on their surface, can mimic the entry of the authentic virus of interest and have been extensively utilized for viral entry research [31]. Pseudovirus-based neutralization assays are a safe and efficient way to measure neutralizing antibodies, as most pseudoviruses are replication-deficient and carry reporter genes. These assays can be easily adapted to emerging viruses by changing the displayed envelope protein, making them a highly flexible and adaptable tool for high-throughput testing [53].

Moreover, their suitability for high-throughput screening makes them efficient in evaluating viral entry inhibitors and assessing preventive measures. One crucial performance indicator for evaluating vaccines is the capacity of vaccine-induced neutralizing antibodies to block viral infection. By utilizing a pseudovirus-based invasion assay, researchers can effectively evaluate the efficacy of RSV vaccines in inhibiting viral invasion. This enables a rapid assessment of vaccine efficacy.

Although pseudovirus-based neutralization assays have been applied to various highly pathogenic viruses, including MERS-CoV, SARS-CoV-2, and EBOV, few studies have been conducted on their application to RSV [38,54,55,56]. One of the most significant reasons for this may be attributed to the membrane fusion capability of F proteins. The RSV F protein mediates viral entry by binding to cellular receptors and triggering fusion between the viral envelope and cell membrane. Several receptors for RSV have been identified, including insulin-like growth factor receptor 1 (IGF1R), intercellular adhesion molecule-1 (ICAM-1), epidermal growth factor receptor (EGFR), and nucleolin (NCL). These receptors are mostly expressed on the pseudovirus packaging cells, HEK293T [57,58,59,60]. When the F protein is expressed, syncytium formation occurs in HEK293T cells [61]. High-level expression of the F protein resulted in increased syncytia formation in HEK293T cells and a significant reduction in the packaging of RSV pseudovirus (Figure 1C,D). Unlike the F protein, which can mediate membrane fusion at neutral pH [61], VSV-G can only mediate membrane fusion at acidic pH levels ranging from 4.8 to 6.4 [62]. This means that the high level of VSV-G expression is less significant in inhibiting pseudovirus packaging than the F protein (Figure 1B). Therefore, it is necessary to identify an optimal level of F protein expression that strikes a balance between efficient packaging of RSV pseudoviruses and syncytium formation. Another reason that has limited the application of pseudoviruses in the study of RSV may be the conformational change of the F protein, which can affect their functionality. The RSV F protein undergoes an irreversible conformational change during membrane fusion, transitioning from a metastable prefusion (pre-F) conformation to a stable postfusion (post-F) conformation. A range of factors, such as fluctuations in temperature, can trigger conformational changes in F proteins [41,63]. Consistent with previous research, our findings demonstrate that thawing at 37 °C preserves pseudovirus infectivity better than thawing at 4 °C, suggesting that a change in temperature triggers a conformational change in the F protein (Figure 2). Preserving the pre-fusion conformation of F proteins is critical for maintaining the infectivity of RSV pseudoviruses or live RSV viruses, as F proteins in the post-fusion conformation cannot induce membrane fusion [19].

In this study, we developed pseudovirus-based assays to evaluate neutralizing antibodies against both subtypes of RSV, namely RSV-A (A2 and Long strains) and RSV-B (B1 strain). To the best of our knowledge, this is the first neutralizing antibody assay against RSV-B subtypes that is based on pseudoviruses. RSV is divided into two subtypes, RSV-A and RSV-B. These two subtypes alternate in circulation at 1- to 2-year intervals, but typically one subtype dominates during each RSV epidemic [64]. The rate of RSV seropositivity in adults over 20 years of age was approximately 100% [65]. Consistent with these findings, our results showed that all healthy adults tested were positive for RSV-A and RSV-B neutralizing antibodies. Additionally, neutralizing antibodies against the RSV-A subtype were significantly higher than those against the RSV-B subtype, suggesting that the RSV-A subtype is more prevalent in this epidemic (Figure 5B). Therefore, the assays established in this study, which can detect neutralizing antibodies against both subtypes of RSV, are powerful tools for epidemiologic and serologic investigations and for screening viral entry inhibitors.

In conclusion, we have developed and optimized a robust lentiviral pseudovirus-based neutralization assay for efficient evaluation of neutralizing abilities against RSV-A (A2 and Long strains) and RSV-B (B1 strain) subtypes. The results obtained from this assay showed high consistency with those obtained from authentic virus neutralization assays. Therefore, this assay can be effectively utilized in various applications, such as studying RSV entry mechanisms, evaluating vaccines, conducting epidemiologic investigations, and screening viral entry inhibitors.

## Data Availability

The datasets generated during and/or analyzed during the current study are available from the corresponding author upon reasonable request.
